# Thermo-mechanical improvement of Inconel 718 using *ex situ* boron nitride-reinforced composites processed by laser powder bed fusion

**DOI:** 10.1038/s41598-017-14713-1

**Published:** 2017-10-30

**Authors:** Sang Hoon Kim, Gi-Hun Shin, Byoung-Kee Kim, Kyung Tae Kim, Dong-Yeol Yang, Clodualdo Aranas, Joon-Phil Choi, Ji-Hun Yu

**Affiliations:** 1 Powder Technology Department, Korea Institute of Materials Science, Changwon, 51508 Republic of Korea; 2 Department of Materials Science and Engineering, University of Ulsan, Ulsan, 44610 Republic of Korea; 3 Department of Mining and Materials Engineering, McGill University, 3610 University Street, Montreal, QC H3A 0C5 Canada

## Abstract

Hexagonal boron nitride-reinforced Inconel 718 (h-BN/IN718) composites were fabricated using a laser powder bed fusion (LPBF) technique to treat a nanosheet-micropowder precursor mixture prepared in a mechanical blending process. Tailoring the BN in IN718 enhanced the thermal resistance of the composites, thereby dampening the sharpness of the melting temperature peak at 1364 °C. This is because the presence of the BN reinforcement, which has a low coefficient of thermal expansion (CTE), resulted in a heat-blocking effect within the matrix. Following this lead, we found that the BN (2.29 g/cm^3^) was uniformly distributed and strongly embedded in the IN718 (8.12 g/cm^3^), with the lowest alloy density value (7.03 g/cm^3^) being obtained after the addition of 12 vol% BN. Consequently, its specific hardness and compressive strength rose to 41.7 Hv_0.5_ **·**cm^3^/g and 92.4 MPa**·**cm^3^/g, respectively, compared to the unreinforced IN718 alloy with 38.7 Hv_0.5_ **·**cm^3^/g and 89.4 MPa**·**cm^3^/g, respectively. Most importantly, we discovered that the wear resistance of the composite improved compared to the unreinforced IN718, indicated by a decrease in the coefficient of friction (COF) from 0.43 to 0.31 at 2400 s. This is because the BN has an exfoliated surface and intrinsically high sliding and lubricating characteristics.

## Introduction

Ni-based Inconel super-alloys are used in the engine manifold of the Space X Merlin rocket engine, which powers the Falcon 9 launch vehicle^1,2^. Moreover, the combustion chamber of the Space X Super Draco rocket engine is manufactured with Inconel using a process of direct metal laser melting, and operates well at high temperature (as high as 650 °C) and pressure (up to 1000 psi)^3^. In particular, Inconel 718 (IN718), based on the NiCrFe austenite γ structure, has long been applied in aerospace machine shafts, bearings, and jet engine turbines due to its strong thermal resistance, high microhardness and strength, and excellent fatigue deformation resistance at high temperature^4–7^. All of these properties are also related to the solid solution formation of second phases (such as the face-centered cubic γ′ structure of Ni_3_(Al,Ti,Nb), the body-centered cubic γ″ structure of Ni_3_Nb, Ni_3_Mo, and carbide phases together with the inclusion of Nb, Mo, and C)^8,9^. Another intermetallic phase frequently present in the IN718 is the orthorhombic δ structure of Ni_3_Nb^10^. However, the anticipated engineering applications of this super-alloy will entail continuous exposure to extreme conditions (high temperature and pressure), which will require a further increase in its specific mechanical strength (absolute mechanical strength, such as tensile and compressive strength, microhardness, shear strength, and so on, divided by density) at high temperature^11–13^. This means an increase in the ability to endure exterior thermo-mechanical force and at the same time, reduction of density to provide a lighter turbine engine system^14^. For this purpose, various reinforcement materials including carbon nanotubes, SiC, WC, TiC, ZrC, TiB_2_, and ZrB_2_ may be considered for incorporation into the Inconel matrix to push its specific thermo-mechanical strength to the maximum^15–20^. However, most of the carbides or borides of the transition metals are synthesized using what are usually expensive methods (such as a self-propagating high-temperature reaction, laser pyrolysis, chemical vapor deposition, and so on) that make them difficult to use in mass production^21–23^. Moreover, some of them exhibit thermal decomposition at high temperature when the Inconel alloy melts, after which desirable properties such as microhardness and wear resistance, deteriorate^20,24^. Furthermore, most of these reinforcement materials also possess relatively high density, and thus are not recommended for aerospace parts; even a 10% weight reduction of the total aircraft can improve fuel economy by 6–8%^25^.

Among many reinforcement materials, hexagonal boron nitride (h-BN, analogous to graphite) has become a promising candidate reinforcement material due to its extraordinary thermo-mechanical properties^26,27^. Of particular interest is the nanosheet form of h-BN, which has high elastic modulus (700–900 GPa) and low density (2.29 g/cm^3^)^28^. Notably, the nanosheets also can act as a thermal shock barrier and prevent deformation of the composites at high temperature^26,28^. Most importantly, the BN reinforcement within the IN718 matrix incorporates a lubricating mechanism that makes the surface more slippery. This provides much improved wear resistance and thus enhanced anti-friction properties of a metal matrix composite (MMC); but only if there is strong bonding and uniform distribution of the reinforcement within the matrix^28–35^. In this respect, the laser powder bed fusion (LPBF) technique combined with a facile blending process allows reinforcement particles to be strongly embedded by the moving laser at uniform intervals in the matrix powders. Compared to conventional manufacturing techniques, the LPBF technique has a wide range of benefits, namely a high level of geometric flexibility, material utilization, and production rate, without using any dedicated tools^36–40^. This technology now appears a very promising method for processing parts made of MMCs^41–45^. Thus, with the application of this system, it is possible to incorporate reinforcement materials readily and strongly by controlling the power and speed of the laser to focus high thermal energy on a layer of powders distributed on a powder bed using a doctor’s blade.

In this work, BN-reinforced IN718 composites with different volume ratios (0, 6, and 12 vol%) of BN were fabricated using LPBF. The resulting phase, microstructure, and mechanical properties were investigated using microscopic imaging, spectroscopic techniques, and mechanical analyses. The morphology and microstructure of the BN-reinforced IN718 composites were compared to those of the standard IN718 alloy to measure the effectiveness of LPBF for the incorporation of BN reinforcement in the IN718 matrix. The spectroscopic results for XRD and XPS provided qualitative information about the chemical state of the MMCs. We also conducted DSC to investigate the thermal behavior of the MMCs, especially to investigate the thermal stability of the IN718 matrix in terms of the phase transformation from a γ″ structure to a δ structure at a temperature of above 650 °C, according to the amount of added BN reinforcement. However, we found that the microstructure in the IN718 region was not as changed as we expected due to the rapid solidification (cooling) effect just after the high energy laser beam irradiation regardless of the amount of added BN. Specific hardness and compressive strength tests (absolute mechanical strength divided by density) were conducted to quantify how strongly the BN reinforcement was bound to the IN718 matrix, and to comprehend the relationship between the reinforcement mechanism and the mechanical strength. Most importantly, the wear resistance mechanism of the composite was investigated based on changes in the COF and wear depth. It was also demonstrated that changes in the surface properties of the BN-reinforced IN718 composites (smoother and more slippery) were related to an increase in BN.

## Results and Discussion

**Figure 1:**
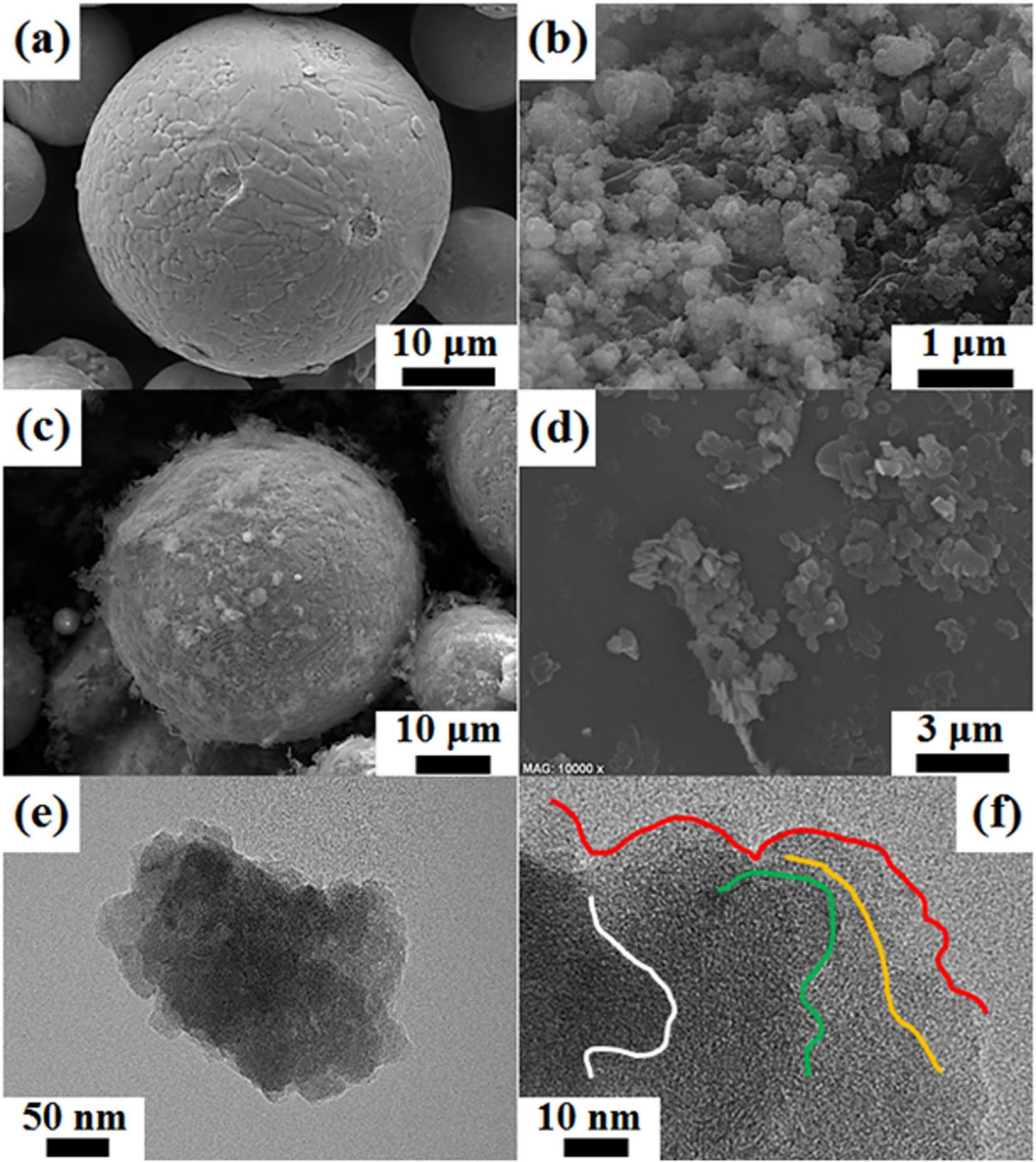
SEM of (**a**) IN718 powders, (**b**) BN clusters (i.e., severely agglomerated in a sheet shape) after the ball-milling process, and (**c**) 12 vol% BN nanosheet + IN718 micropowder mixture. SEM of (**d**) raw BN sheets before the ball-milling process. Low magnification TEM of (**e**) BN nanosheets dispersed after ball milling followed by harsh sonication. High-resolution TEM of (**f**) BN nanosheets with several (about 7–8) interplanar layers.

As shown in Fig. 1a, the gas-atomized IN718 micropowders were mainly spherical. BN clusters (Fig. 1b) highly agglomerated in the nanosheets were fragmented into smaller pieces during the ball-milling process^28^. According to Lee’s research, when they repeated the fracturing and functionalizing processes of h-BN sheets through the planetary ball-milling process with a subsequent centrifuging process, the sheets were transformed to a nanosheet shape with an exfoliated structure^28^. In our research, however, more cotton-like, aggregated powders were first formed just after the ball-milling process due to the significant agglomeration according to the strong van der Waals force among the BN nanosheets^28^. Fortunately, the BN clusters were relatively well separated by applying very harsh sonication followed by centrifugation, after which they once again acquired a nanosheet shape. Figure 1c shows IN718 micropowders mixed with 12 vol% BN nanosheets. Some BN nanosheets were still stacked and agglomerated around the IN718 powders caused by BN clusters once again recombining into BN nanosheets despite the severe blending process. In fact, the BN nanosheets were fabricated by the raw BN sheets (Fig. 1d) being subjected to the ball-milling process with subsequent sonication^28^. Figure 1e shows a TEM image of the more exfoliated BN nanosheets despite them still having a slightly agglomerated shape and a lateral size of several hundred nanometers. However, there were only several distinctive interplanar layers in the internal space of the exfoliated BN nanosheets; thus, we determined that the high mechanical energy of planetary ball-milling and subsequent harsh sonication processes were useful for the fabrication of the few-layered BN nanosheets. As shown in Fig. 1f, when HRTEM observation is used, the number of layers of the fabricated BN nanosheets can be directly confirmed by counting the interplanar lines at the edge of the BN nanosheets. That is important because different layers of BN nanosheets exhibit different thermal behavior, and furthermore their physical, chemical, and mechanical properties are highly related to the number of layers^28^. In particular, there is a linear relationship between the number of layers and the absolute hardness of BN nanosheets^46,47^. However, we found that the absolute hardness of the BN-reinforced IN718 composites was not related to the number of layers of BN nanosheets because the BN nanosheets were agglomerated and recombined again during the blending process, and most of the BN aggregates were directly consolidated through the high energy of laser irradiation regardless of the number of layers of BN nanosheets. Otherwise, the absolute hardness of the composites is highly related to the fraction (concentration) of the added BN nanosheets due to the intrinsic low microhardness of BN^28^. Comparatively, the wear resistance of the IN718 composites reinforced with the BN nanosheets was improved because the composites experienced better lubrication and sliding with a larger amount of well-dispersed BN reinforcement. This was caused by the BN nanosheets being consolidated by the high energy of the laser irradiation in the IN718 matrix regardless of the degree of exfoliation in the former.

Particle size analysis of IN718 micropowders revealed a narrow size distribution with a mean diameter (D_50_) of 34.5 µm, while ball-milled BN nanosheets had D_50_ of 0.2 µm, as shown in Supplementary Figure 2a and b, respectively. Thus, the BN/IN718 nanosheet/micropowder mixture had a D_50_ of 28.3 µm due to the presence of BN nanosheets between the IN718 micropowders, as shown in Supplementary Figure 2c. The size of the BN nanosheets was much smaller than the diameter of the IN718 micropowders, thus the BN nanosheets effectively entered the spaces between the IN718 micropowder particles during the blending process before they were irradiated with the laser beam^48–52^. This behavior is in accordance with theory for bimodal systems (nanosheets and micropowders)^48–52^. After this, it was possible to achieve adequate laser fusion of the points of increased contact between the nanosheets and micropowders. Overall, the secondary phase BN reinforcement can be uniformly dispersed and strongly embedded in the IN718 matrix.

**Figure 2:**
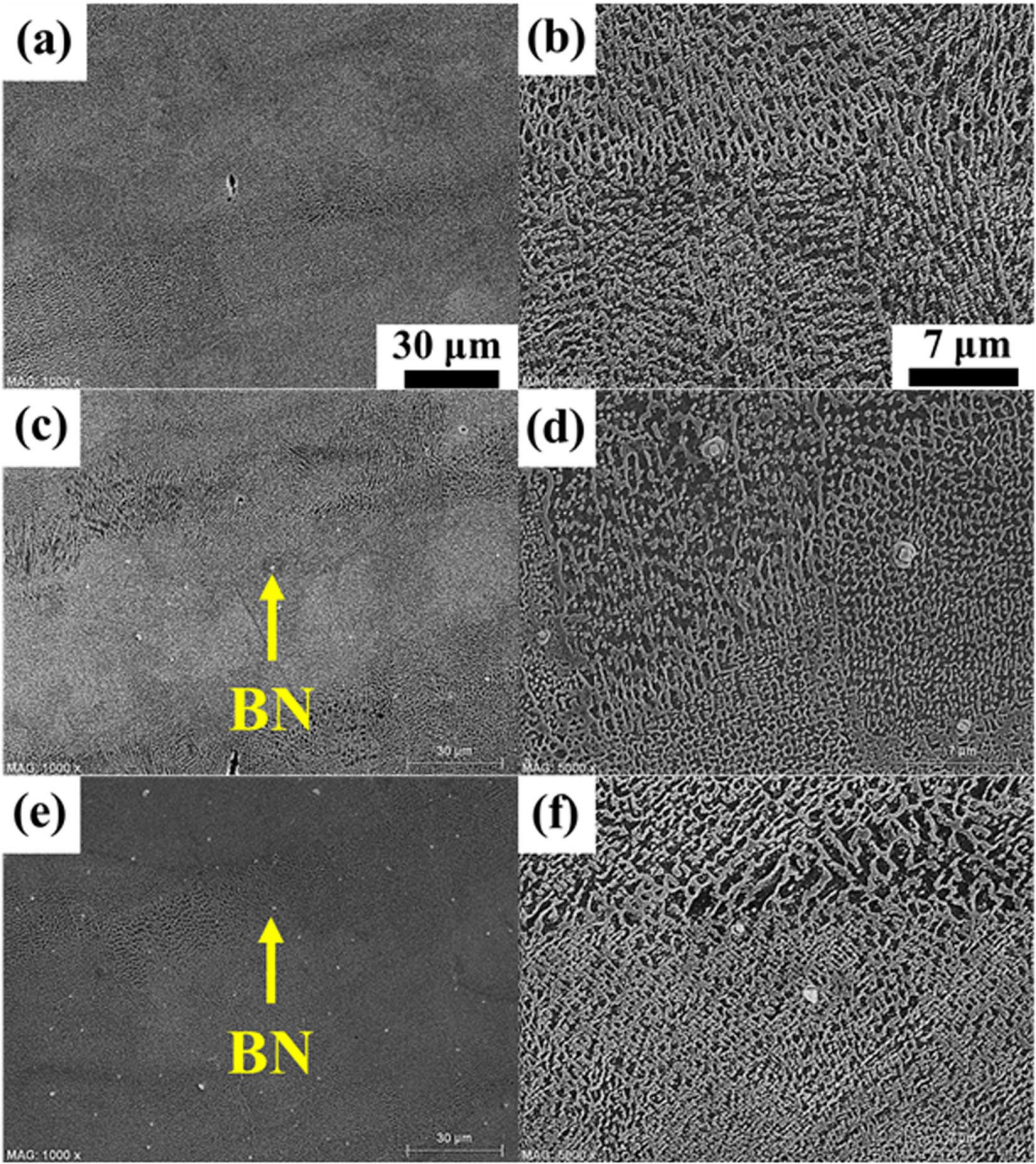
Cross-sectional SEM of (**a** and **b**) IN718 alloy, (**c** and **d**) IN718 composite reinforced with 6 vol% BN, and (**e** and **f**) IN718 composite reinforced with 12 vol% BN after polishing and etching.

The BN-reinforced IN718 composites were polished and etched so that through the cross-sectional SEM analysis, we were able to observe the phase transformation of the intercellular structure of IN718 caused by the increased amount of BN reinforcement. Figure 2 shows the cross-sectional microstructure of the SLM-processed IN718 alloy and composites with different BN reinforcement content (0, 6, or 12 vol%). As seen in these micrographs, the microstructure of the alloy and composites, after conducting LPBF, was very dense. This was due to >95% relative density of the alloy and composites without the formation of critical deficiencies. In particular, the SEM images in Fig. 2a and b exhibited fine dendritic structures (i.e., elongated and cellular structures). This was because of the horizontal heat flux in the cross-section of the Inconel alloy, which was related to the movement of the laser heat source. These microstructures typically occur during the SLM process, induced by the high thermal energy density and rapid solidification.

After the powder bed processing of the nanosheet-micropowder mixture, the high energy of the laser irradiation toward the BN clusters with the IN718 powders provided a more voluminous, fused form of BN reinforcement in the IN718 matrix. In fact, the BN reinforcement embedded in the IN718 matrix should have been more abundant, which would have meant high detection density of functionalized BN reinforcement in IN718 matrix, as shown in Supplementary Figure 3. However, the large amount of BN reinforcement consolidated in the IN718 matrix was detached or omitted regardless of the bonding strength between the two materials due to the vigorously conducted polishing and strong acid etching processes, as shown in the cross-sectional images of the composites. Consequently, much less abundance (density) of the BN reinforcement was found in the IN718 matrix than should be there.

As seen in Fig. 2c and d, the BN reinforcement was uniformly dispersed throughout the IN718 matrix. Interestingly, the positive effect in terms of the thermo-mechanical properties of the MMCs was mainly related to the dense laser heat treatment at high power, which even transformed the morphology of the BN from an irregular nanosheet to a smoother, denser, more spherical shape, and at the same time, tightly incorporated the BN reinforcement within the IN718 matrix. In fact, this can lead to instability of the melt pool, and might have a significant influence on the bonding of micropowders and/or nanosheets between neighboring laser scan tracks or layers. Furthermore, this can decrease the density of the composites by creating thermal balling, interlayer pores, and mechanical cracks. However, this phenomenon was not significantly observed with the SLM-processed MMCs, which indicated that these amounts of BN reinforcement (i.e., 6–12 vol% BN) could be incorporated into the IN718 matrix with acceptable results.

As shown in Fig. 2e and f, the laser irradiation to the powder bed of the nanosheet-micropowder mixture generated high densification (>95%) of the composites and thus increased their thermo-mechanical stability, especially until the content of the BN nanosheets increased up to 12 vol%. Meanwhile, the irregularly aggregated BN clusters changed to a more dome shape by the high energy laser irradiation because some BN nanosheets were highly stacked by a strong van der Waals force, despite the significant mechanical blending process^28^. In fact, the original surface images of BN-reinforced IN718 composites should look like those in Supplementary Figure 3 when they are fabricated without any post-processes (i.e., polishing and/or etching). However, when we conducted vigorous polishing and strong acid etching processes so as to be able to examine the cross-sectional images of the MMCs, there was much less BN reinforcement (low BN reinforcement density) because it was detached and omitted from the IN718 matrix regardless of the bonding strength between the two materials. Consequently, there was less BN reinforcement in the IN718 matrix than we expected.

In order to identify the presence of the BN reinforcement embedded in the IN718 matrix after the LPBF process, X-ray spectroscopic analyses such as EDS mapping, XRD, and XPS were conducted. In particular, EDS mapping images of the BN-reinforced IN718 composites showed that most of the BN clusters (Supplementary Figure 5) were transformed into their consolidated bulk state. XRD patterns of the composites indicated that the BN reinforcement maintained the hexagonal structure, which means there was no thermal and/or chemical decomposition of BN. In the XPS results of the composites, a clear peak presented at 190.8 eV in the lower peak of the B 1 s band, which indicated the presence of BN. Thus, we confirmed that the BN reinforcement was maintained in the IN718 matrix despite the high energy of the laser irradiation.

The cross-sectional microstructures of the laser-irradiated surfaces of the BN-reinforced IN718 composites showed the relatively well dispersed BN reinforcement in the IN718 matrix. However, the intercellular structure of the IN718 did not vary sufficiently to show any noticeable tendency except for the increased BN detection density embedded in the IN718, as analyzed from the obtained SEM images. This was because the sudden cooling process provided rapid solidification (10^3^–108 K/s) in the composites after laser beam irradiation of the thinly spread powder bed of the nanosheet-micropowder mixture over a very short time period^53^. Following this, the microstructure of the IN718 region was not changed much in spite of the high energy of laser beam irradiation. However, the BN form became more consolidated. Overall, more BN reinforcement was found in the IN718 matrix as the amount of the added BN nanosheets increased to 0, 6, and 12 vol%, although the microstructure of the IN718 region did not change much due to the rapid solidification effect, regardless of the amount of added BN.

**Figure 3:**
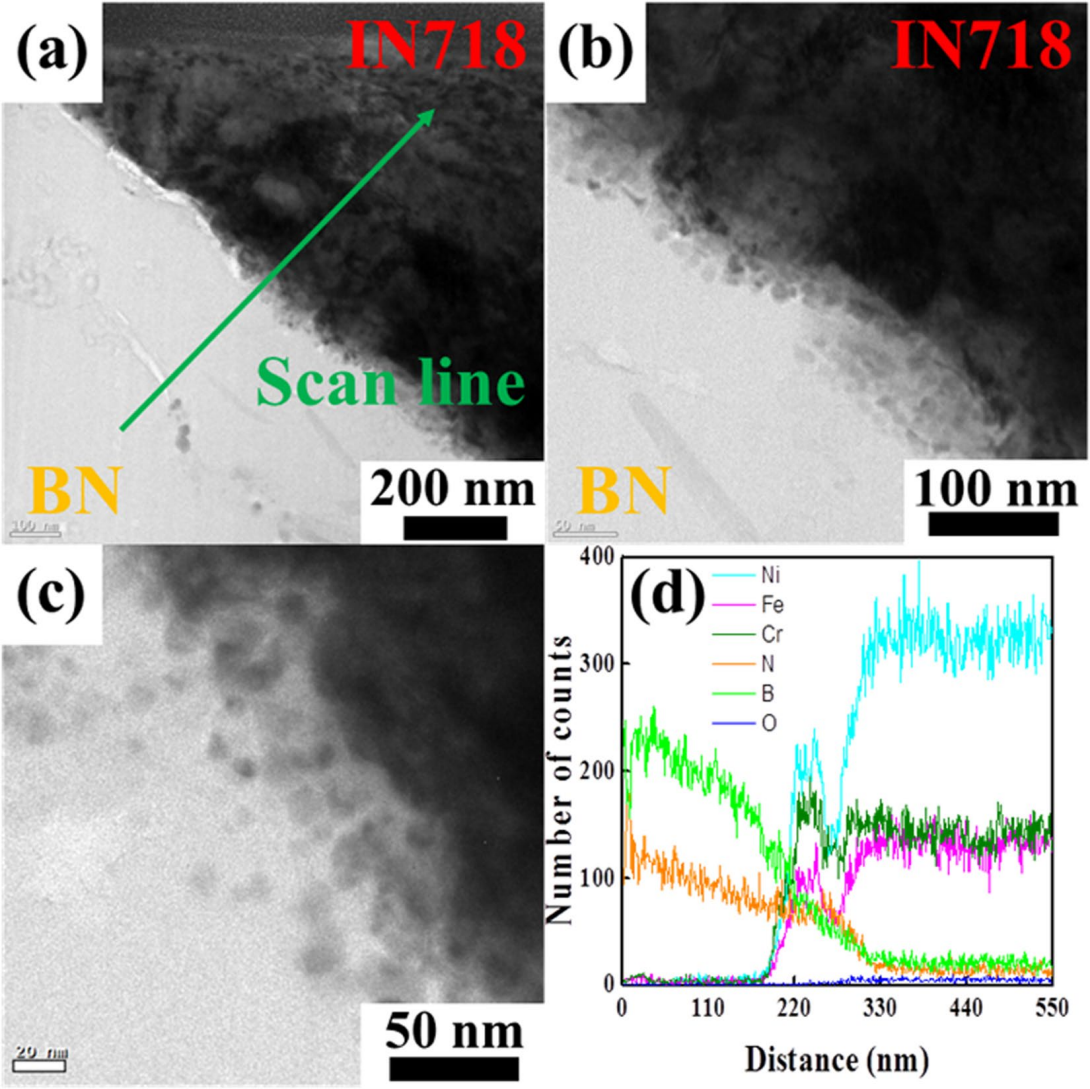
Conventional TEM images (**a**–**c**) and EDS scan profile (**d**) across the BN/IN718 interface in (**a**) along the green line of the 12 vol% BN-reinforced IN718 composite.

The microstructural examination of the 12 vol% BN-reinforced IN718 composite was extended using TEM. Figure 3a–b shows low-magnification TEM images of the MMC. In these images, the BN reinforcement (the lighter area) was retained within the IN718 matrix (the darker area) with a clean interface, containing no voids, cracks, or other defects. This indicated strong interfacial bonding. At the same time, small spherical particles of IN718 were found in the wetting system at the interfacial boundaries between BN and IN718, as shown in Fig. 3c, because the high energy of the laser beam irradiation promoted partial melting of BN and completely melting of IN718 at their interfaces. For the proof of this, the presence of particles randomly aligned close to the interfacial boundary represented the high diffusion reaction of IN718 from use of the SLM process. That is to say, work in progress shows that the BN phase may serve as a preferred nucleation site for the adjacent IN718 phase according to the stimulated nucleation effect^54–56^. Whatever nucleation of IN718 was envisaged, the stabilizing energy at the interfacial boundaries of the BN made the occurrence of random orientation of IN718 particles very limited in interactions between BN and IN718^54–56^. Namely, the completely molten IN718 percolated into the partially molten BN during the diffusive migration of IN718; then solidified rapidly on contact with the partially melted BN^54–56^. Overall, the alignment of the IN718 particles shows that there was high diffusion and sudden cooling at the interface between BN and IN718, which corresponded to the unique characteristics of the laser heat treatment. However, even with such a highly diffusive process, there was no thermal degradation or chemical reaction of BN with the constituents of IN718, as was demonstrated by SAED pattern analysis. The alloying elements of completely molten IN718 (mainly NiCrFe intermetallic compound) were diffused into the partially molten BN, but with limited solubility. In fact, Li *et al*. previously reported restrictive diffusion of the ceramic reinforcement in the metal matrix during the SLM process, because there were only limited interfacial interactions between the metal and the ceramic^57^. To evaluate the chemical composition of the BN/IN718 composite, an EDS line scan across the interface was also performed, as illustrated in Fig. 3d. The lined arrow (perpendicular to the interface) shows two types of composition: BN phase with high amounts of boron and nitrogen, and IN718 phase with high amounts of Ni, Cr, Fe, and Nb. There was also oxygen all over the area. These results confirmed that the main elements typical of BN and IN718 were distributed well away from the interface.

**Figure 4:**
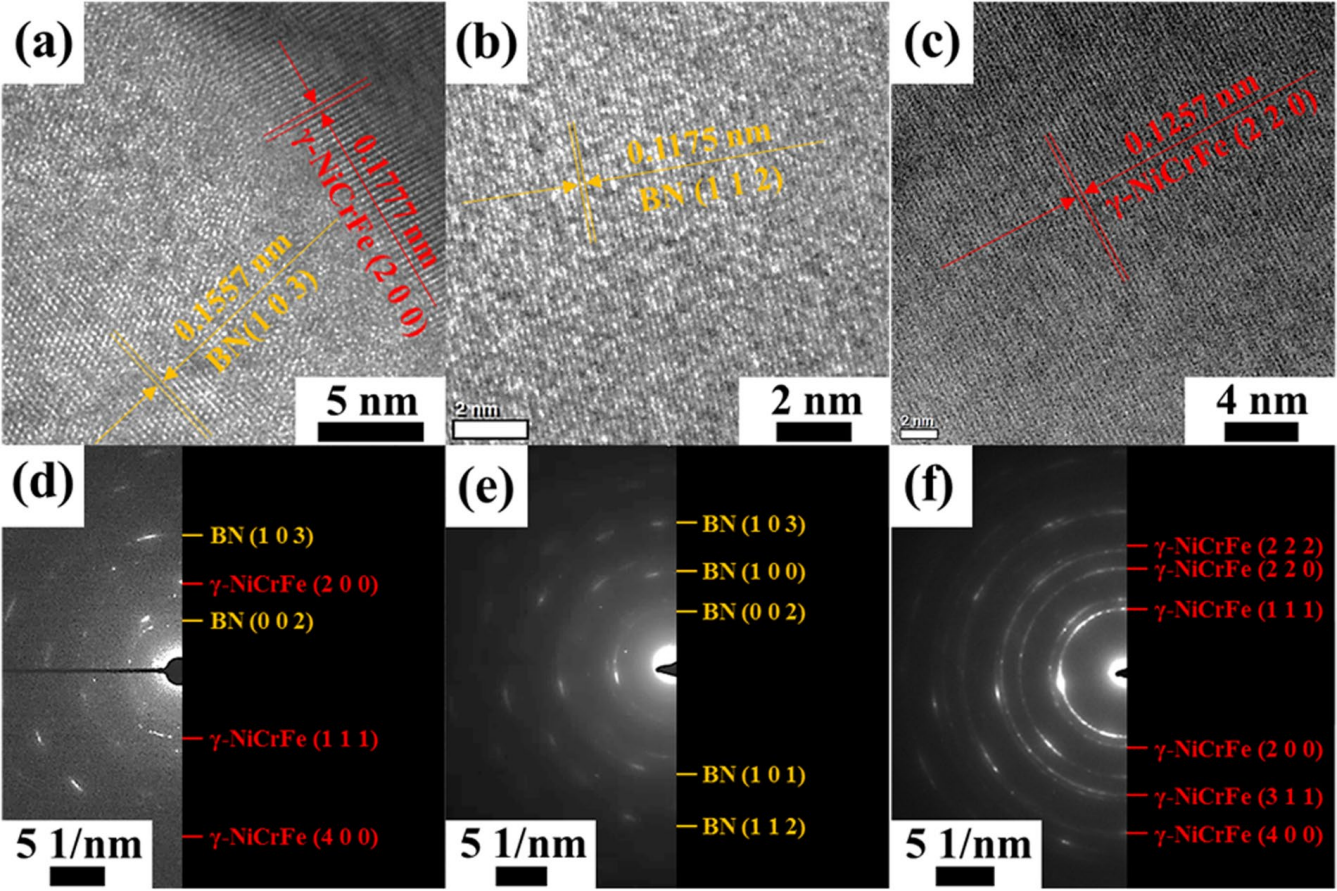
High-resolution TEM images (**a**–**c**) with lattice fringe spacing measurements (**a**) interface region between BN and IN718, (**b**) BN region, and (**c**) IN718 region), and corresponding SAED patterns (**d**–**f**) of IN718 composite reinforced with 12 vol% BN.

Figure 4a shows a high-resolution TEM image of the tangled particles accumulated at the interface between BN and IN718. The lattice fringe measurements in such a region revealed the presence of overlapping BN and IN718 (Fig. 4b of BN compared to Fig. 4c of IN718). The SAED pattern (Fig. 4d) at the bi-material interface also exhibited a combination of the two patterns (BN and IN718). All three SAED patterns conformed to a ring pattern, which explained the small grain size of the molten NiCrFe phase adjacent to the partially melted BN phase in the SLM-processed composite. However, no new peaks appeared, which indicated that there was no chemical reaction between BN and IN718 (i.e., impurities or oxides), during the high-energy laser melting and consequent rapid solidification. As a result, SAED patterns were taken at the interface and exhibited continuous ring patterns corresponding to the hexagonal structure of BN and the face centered-cubic structure of NiCrFe. These indicated that no diffusion reaction was found in the interfacial region between the BN reinforcement and IN718 matrix.

**Figure 5:**
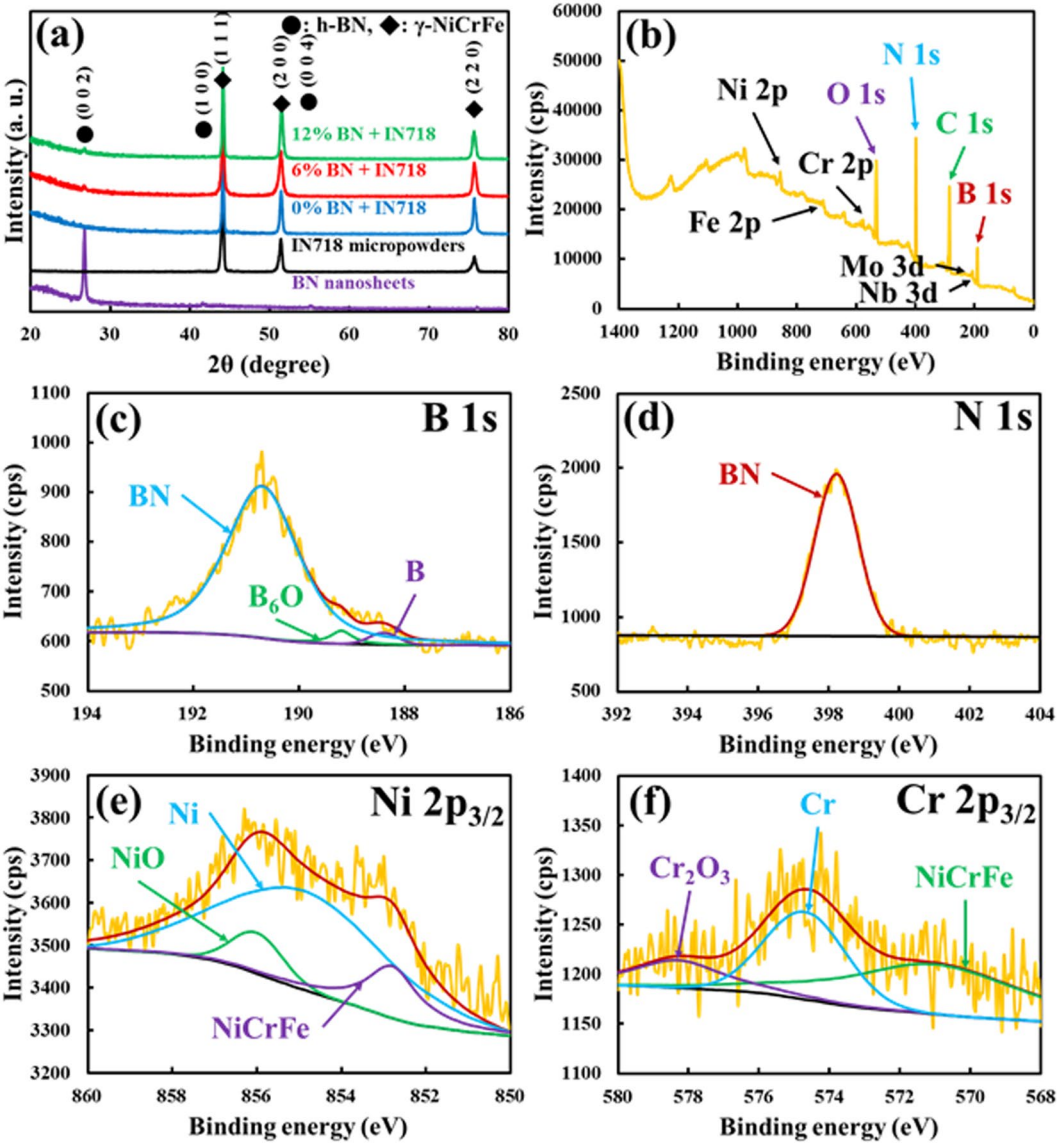
XRD (**a**) of BN nanosheets, IN718 micropowders, IN718 alloy, and IN718 composites reinforced with BN. XPS (**b**–**f**) of IN718 composite reinforced with 12 vol% BN.

As shown in Fig. 5, the crystalline structures of the SLM-built alloy and composites were analyzed using their XRD patterns to ascertain which phases were present, and to compare the crystalline structures of the individual BN nanosheets and IN718 micropowders. Initially, IN718 had a face-centered cubic structure similar to that reported in other work^4,6^. There was also the presence of the reinforcement in the matrix, which was demonstrated by a variation in the peak position at 2θ = 26.6°, corresponding to a (0 0 2) crystal plane of BN with a hexagonal structure^28^. Because the agreement in the relative peak position of the XRD was indicative of the state of the reinforcement in the matrix, this determined that the BN reinforcement was chemically stable and maintained its original state within the IN718 matrix. Specifically, it appears that during the SLM process, the BN reinforcement was partially melted within the completely molten IN718 matrix; but, there was neither chemical reaction of BN reinforcement with the IN718 matrix, nor generation of new metal nitrides, from the constitutional elements of IN718 and the nitrogen of BN during the diffusion process. Because the heating and cooling rates in the SLM were very high, and the diffusion process of precursors occurred over a very short interval of time, the BN was partially melted and then quickly stabilized close to the IN718. This mechanism was consistent with the results in a reference in which a nano-TiB_2_/AlSi10Mg composite was produced by SLM using an AM method^57^. Based on the thermodynamic data between boride and nitride compounds of BN reinforcement and the constitutional elements (Ni, Cr, Fe, and so on) of the IN718 matrix, the weak diffusion of boron from BN did not result in production of any boride compounds, even though high thermal energy was applied to both of the precursors. This was because the activation energy barrier of boron and nitrogen in BN was too high to break and create new bonds with the elements of IN718^26^. For the same reason, no new metal nitrides were formed from reaction between nitrogen in BN and the elements constituting IN718. As a result, there was no chemical reaction between BN and IN718. The energy imparted by the selective laser was insufficient to bread the bond between boron and nitrogen, preventing creation of new bonds with the other elements (Ni, Cr, Fe, and so on) in IN718, and with boron or nitrogen (such as nickel boride, chromium boride, iron boride, or nitrides). However, there was enough thermal energy to partially melt BN and to completely melt IN718.

As shown in Fig. 5, XPS analysis was also performed to more precisely investigate the chemical states of each elemental constituent in the SLM-built IN718 composite reinforced with 12 vol% BN. The survey spectrum (Fig. 5b) and high-resolution spectra of B 1 s, N 1 s, Ni 2p_3/2_, and Cr 2p_3/2_ were recorded, and chemical compositions from those analyses were established. To be specific, the high-resolution spectra of B 1 s and N 1 s were provided here for clarity about the presence of BN, which was not clearly shown in the XRD analysis. The corresponding deconvolution peaks of BN, with specific binding energies, are reported in Fig. 5c and d. Initially, the B 1 s spectrum consisted of a triplet signal separated by one major peak of BN at 190.8 eV, and two minor peaks of B_6_O and B^0^ at 189.2 and 188.4 eV. The N 1 s spectrum consisted of one broad peak, which was assigned to the BN compound (N 1 s = 398.3 eV). Then, the Ni 2p_3/2_ spectrum was represented in Fig. 5e, and it was determined that the Ni content was the greatest portion (50.1 wt%) found in any of the elements. In particular, the Ni 2p_3/2_ spectrum exhibited a main peak at 855.2 eV, and two supplementary peaks at 856.2 and 852.9 eV. The presence of a main peak together with side peaks (skewed) resulted from interactions between Ni and O, or other elements (Cr and Fe). As shown in Fig. 5f, the Cr signal can be detected in each of the Cr_2_O_3_, Cr, and NiCrFe peaks. Although Cr in IN718 strongly interacted with Ni and Fe, the original phase of the Cr^0^ core peak was mainly retained. The C 1 s signal observed in the survey spectrum was associated with C in IN718 mainly due to its tendency to form metal carbides. Meanwhile, the O 1 s signal at 532.0 eV can be correlated with formation of metal oxides after atmospheric exposure of the samples.

**Figure 6:**
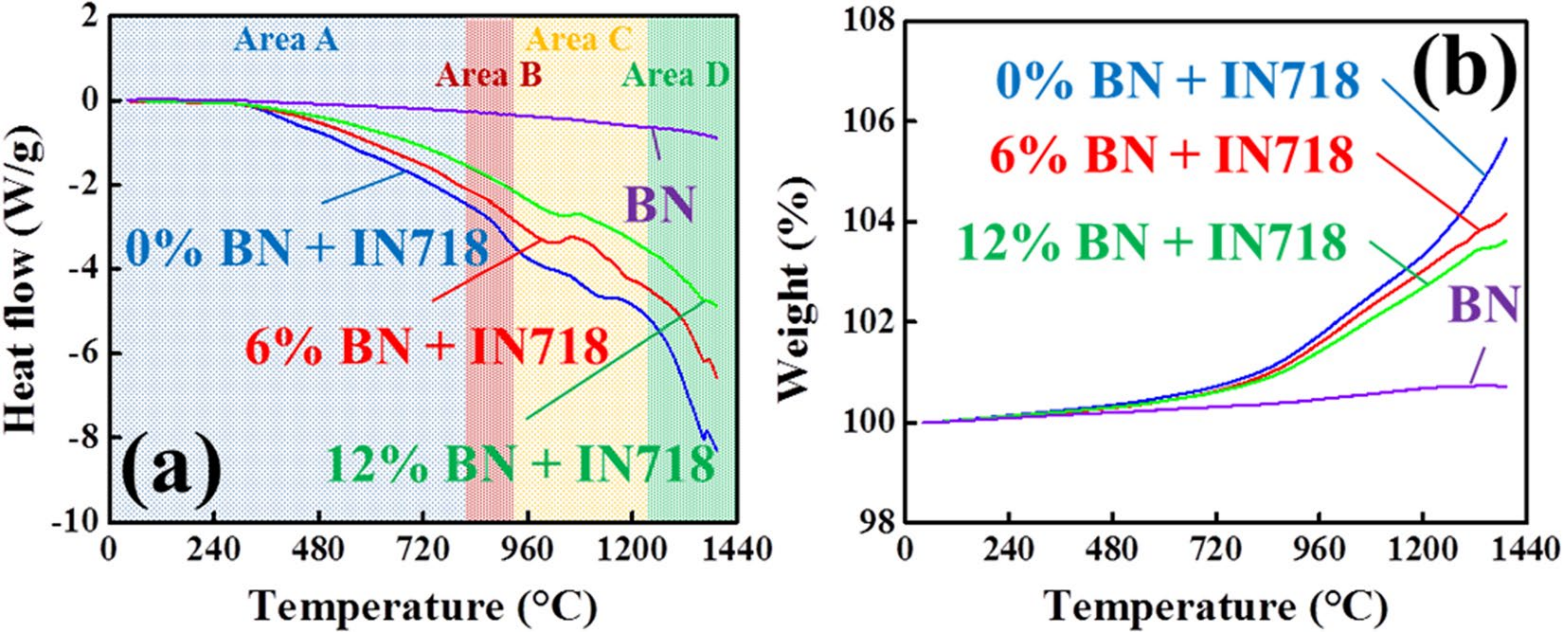
DSC (**a**) and TGA (**b**) of BN nanosheets, IN718 alloy, IN718 composite reinforced with 6 vol% of BN, and IN718 composite reinforced with 12 vol% BN.

The transformation of intermetallic phases of IN718 according to the temperature has been extensively studied; it is generally known that the main precipitates that contribute to mechanical strength of IN718 at high temperature are the body-centered cubic coherent γ″ [Ni_3_Nb] phase and the face-centered cubic coherent γ′ [Ni_3_(Al, Ti)] phase^10,20,58^. In particular, the γ″-phase is the major strengthening phase^10,20,58^. Another intermetallic phase formed in the super-alloy is the orthorhombic incoherent δ [Ni_3_Nb] phase^10,20,58^. The γ″-phase precipitates between about 600 °C and 900 °C, and the δ-phase emerges at temperatures from 750 °C to 1020 °C^10,20,58^. With an increase in temperature or longer aging time, the γ″-phase can coarsen or transform to the equilibrium δ-phase^10,20,58^. Such change in the microstructure influences the mechanical and thermal properties of the alloy due to the nature, morphology and formation kinetic of the δ-phase, which sets the temperature-time limits for various high-temperature applications of IN718^10,20,58^. Figure 6a shows the DSC results of the alloy and composites up to 1400 °C, with a heating rate of 10 K/min. To be specific, the heating curve (blue line) of the IN718 alloy exhibited three pronounced peaks at (804, 928, and 1173) °C, and a sharp peak at 1364 °C^10,20,58^. First, the exothermic peak at 804 °C (area A) was related to the γ″-phase precipitation. The second exothermic peak at 928 °C (area B) corresponded to the δ-phase transformation, with dampening by 12 vol% BN reinforcement with a very low CTE (1–4 × 10^−6^ °C^−1^) and thus a thermal-shock-barrier effect^10,58^. Following this transition peak, the endothermic peak corresponding to a temperature of 1173 °C (area C on the heating curve) indicated the melting temperature of the Laves/γ compound and the precipitation of NbC^20^. The Laves/γ compound is a Nb- and Mo-rich phase at the dendrite surface region^20^. In addition to the similarity at the early stage of the BN-reinforced IN718 composites, the peak became smoother and more ambiguous as the amount of BN increased. Finally, the sharp peak at 1364 °C (area D) was the melting temperature peak, which was attributed to the solid-liquid transition. However, the solid/liquid transition peak of the composite reinforced with 12 vol% BN declined considerably because the high thermal resistance of BN significantly disrupted the diffusion of IN718. This also agreed well with the TGA results presented in Fig. 6b and justified the relation shown (in the thermogram) between the indistinctive melting temperature of the composites and the amount of reinforcement added. For the distinguishable TGA curve of the 12 vol% BN-reinforced IN718 composite, the small dips around 1364 °C had no pronounced peaks due to the artefact caused by thermal inertness and the contribution of the thermal shock barrier effect of BN. As a result, we determined that the heat resistance was significantly improved by reinforcing IN718 with BN.

**Figure 7:**
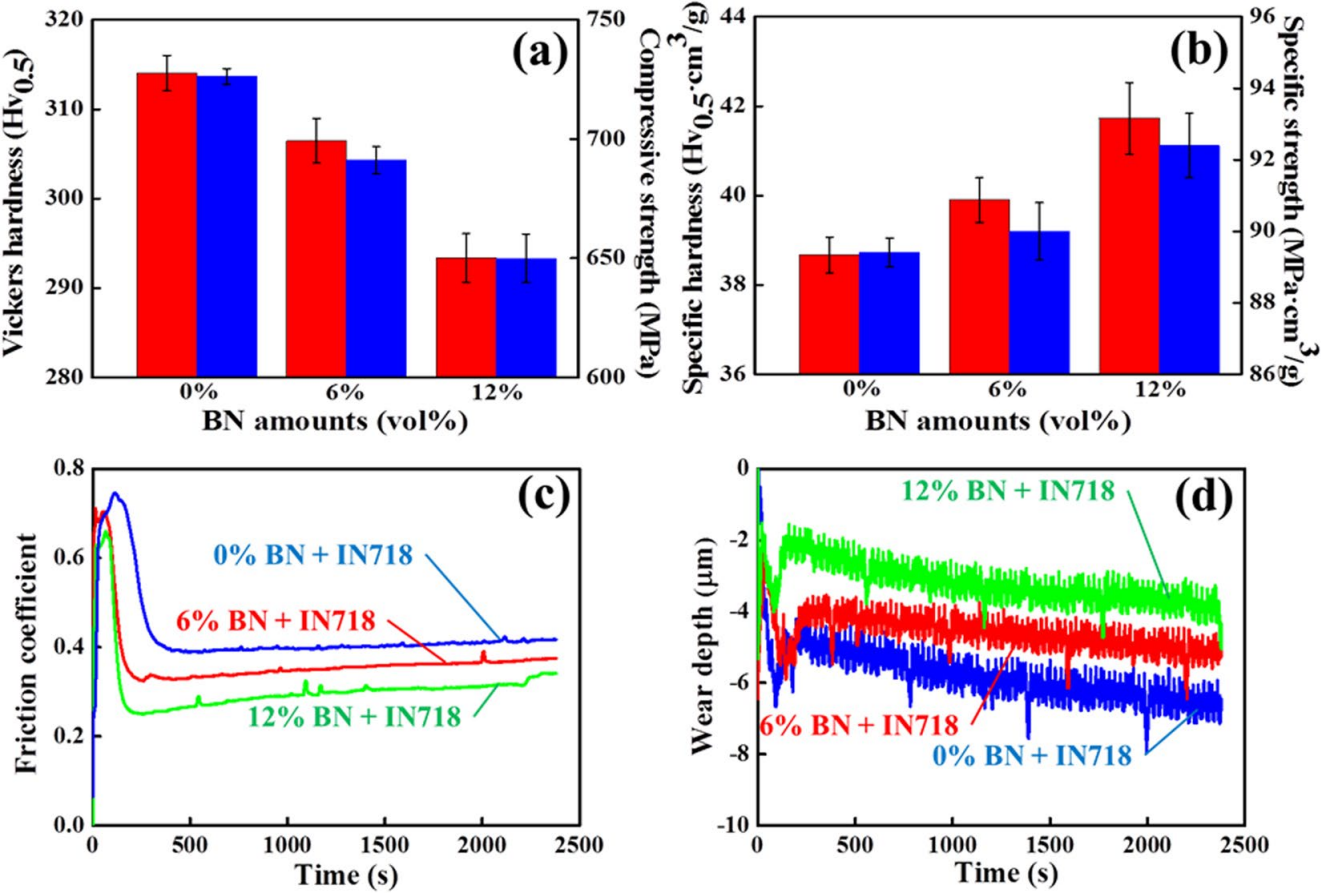
Comparison of Vickers hardness (left bar in red) and compressive strength (right bar in blue) (**a**) and specific hardness (left bar in red) and compressive strength (right bar in blue) (**b**) of IN718 alloy, IN718 composite reinforced with 6 vol% BN, and IN718 composite reinforced with 12 vol% BN. Friction coefficient (**c**) and wear depth (**d**) as a function of the amount of BN used to reinforce the IN718 matrix.

The decline in the relative density, and increase in the specific hardness and compressive strength of the composites, in relation to the increase in the amount of BN reinforcement are recorded in Table 1 and Fig. 7. As shown in Fig. 7a, the SLM-processed, standard IN718 alloy displayed a microhardness of 314.1 Hv_0.5_. After reinforcement with 6 vol% BN, the microhardness slightly decreased to 306.5 Hv_0.5_ while the reinforcement of IN718 with 12 vol% BN resulted in much lower microhardness of 293.4 Hv_0.5_. In fact, this decrease in microhardness was related to an intrinsic property of the BN reinforcement (low microhardness), although the SLM process induced a composite with relatively strong bonding between BN and IN718. As shown in Fig. 7a, the compressive strength (726.3 MPa) of the standard IN718 alloy was also comparable to that of the IN718 composite reinforced with 6 vol% BN (gradually decreased to 691.1 MPa). The compressive strength then decreased even more with addition of 12 vol% BN reinforcement (649.9 MPa). Based on this observation, an approximate 6.4 MPa decrease in compressive strength is expected per 1 vol% addition of BN. This incremental decrease in the compressive strength measurement of the composites was mainly related to the heterogeneous bonding between the ceramic BN and the metallic IN718. Overall, the decrease in the microhardness of the composites commenced with an increase in the BN amount from 0 to 12 vol%. As similarly shown for microhardness, the compressive strength also decreased for the IN718 composite with 12 vol% BN reinforcement. These decreases in microhardness and compressive strength can possibly be attributed to the intrinsic low microhardness of BN, and to the heterogeneous conjugation between BN and IN718 inheriting the low wettability of the ceramic and the metal, thus causing the formation of slightly more pores and cracks (mechanical defects). However, the specific hardness and compressive strength (Fig. 7b) of the MMCs incrementally increased from 38.7 Hv_0.5_ **·**cm^3^/g and 89.4 MPa**·**cm^3^/g to 41.7 Hv_0.5_ **·**cm^3^/g and 92.4 MPa**·**cm^3^/g, respectively, due to the relatively strong bonding between the ceramic BN and the metallic IN718. Specific mechanical strength is defined by absolute mechanical strength, such as tensile and compressive strength, microhardness, shear strength, and so on, divided by density. The specific mechanical strength of the composites gradually increased with the increased content of BN reinforcement although the absolute hardness and compressive strength slightly decreased due to the addition of BN ceramic with intrinsic low microhardness and compressive strength. This was also because the reduction of the overall density of the composites according to the addition of BN with a very low density of 2.29 g/cm^3^ was much more significant (much more abruptly decreased) than the absolute mechanical strength decrease. That is to say, this was due to the high thermal energy of the laser beam with regard to the substantial decrease in relative density caused by the addition of 12 vol% BN with a low density of 2.29 g/cm^3^. Therefore, the estimated increase in specific hardness and specific strength per 1 vol% addition of BN is about 0.3 Hv_0.5_ **·**cm^3^/g and 0.3 MPa**·**cm^3^/g, respectively. Figure 7c and d shows the COF and wear depth for unreinforced IN718 alloy, and for composites with 6 or 12 vol% BN, under a load of 5.0 N with a WC bead. Initially, the COF of standard IN718 increased abruptly from the initial value of 0.07 to maximum 0.74 within 500 s due to increase in the contact area, and then fluctuated around 570 s until the end of the test, as shown in Fig. 7c. The high COF of the unreinforced IN718 alloy caused extensive abrasion and scratching (its standard wear resistance mode) which made the contact area undergo a pulling-out junction with high shear force. On the other hand, slippery and lubricating properties resulting in a lower COF was predominant in the BN-reinforced composites during the same tribology test. After the surface layer was worn enough by the same WC bead, which started to expose the inside of the composite, it provided a true value for the COF. The COF was highly affected by the shear strength, which was explained by the wear mechanism of the composites. Thus, the COF was clearly disproportional to the amount of exfoliated BN reinforcement. More specifically, first, the unreinforced IN718 alloy had the highest COF compared with all of the composites incorporating BN nanosheets. Then, for the 6 vol% BN-reinforced IN718 composite, the increase in the sliding frequency and lubricating behavior gave rise to reduced friction between the sample and bead, thereby decreasing the COF. Finally, the COF dramatically decreased for the composite containing 12 vol% BN, leading to less frequent grooves during the time of application. Overall, the increase of exfoliation from the BN reinforcement with an increase from 0 to 12 vol% established a definite reinforcement mechanism. This can be attributed to the significant lubricating behavior of the BN reinforcement via its hexagonal structure and surface exfoliation, which greatly decreased abrasion of the IN718 matrix and led to an increase in wear resistance. As can be seen in the wear depth of the alloy and composites (Fig. 7d), the wear mechanism was explained by the reinforcement effect; however, this process rarely occurred in the IN718 alloy due to the absence of BN. To be specific, it could be observed that the standard IN718 alloy had relatively weak wear resistance, and the sample was distinctly scratched and abraded. Thus, the wear depth of the IN718 was also between 0.0 and −6.6 µm within the initial 500 s, increased gradually to −4.9 µm, and then fluctuated during the rest of the sliding time. The initial high value was attributed to the intrinsic wear behavior of IN718, which is related to the surface oxidation of Ni, Cr, and Fe elements, and/or the intermetallic compounds of the IN718 alloy. Thus, from the graph of the wear depth of the IN718, severe grooves and high resulting wear depth were analyzed, showing a combination of abrasive wear force and shear resistance force. These results indicated that the unreinforced IN718 alloy was not effective at maintaining strong wear resistance against highly abrasive friction. With the addition of 6 vol% BN to IN718, the wear depth of the BN/IN718 composite was less than that of the IN718 alloy, which confirmed the occurrence of the reinforcement effect in this composite. This was due to the simultaneous effect of both the reinforcement by BN nanosheets and their relative greater sliding and lubricating effect. Previously, Lee *et al*. determined that the high amount of reinforcement on the sliding surface resulted in less scratching (anti-friction), leading to less loss of the Si_3_N_4_ matrix material from the worn surface in the reinforcement form of BN flakes^28^. In practice, the average wear depth of the composite after reinforcement with 6 vol% BN was −5.1 µm during the entire 2400 s process. The composite had a relatively stable and much lower wear depth, in contrast with the unreinforced IN718 alloy. Finally, the wear depth of the composite reinforced with 12 vol% BN was obtained. From this, it was clear that this composite had the shallowest wear depth, even though there were high initial fluctuations in both measurements. As previously shown in the COF, the wear depth also rapidly became stable after 500 s. As a result, the final wear depth of the IN718 composite with 12 vol% BN reinforcement was more than 22.7% and 40.9% (the wear depth for unreinforced IN718 alloy and for 6 vol% BN-reinforced composite, respectively) because the wear resistance of the composites effectively improved with increase in the amount of BN reinforcement. That is to say, the lubricating property of the BN reinforcement not only improved the COF of the wear surface, but also decreased the wear depth. Overall, the BN sheet itself has an exfoliated structure, and surface fragments in the layers adjacent to the IN718 provided an additional sliding effect due to the presence of the reinforcements between areas of the harder surface of the matrix. This also indicated that the lubricating property of the BN reinforcement not only improved the COF of the wear surface, but also decreased the wear depth. Thus, the composites reinforced with BN contributed to better anti-friction and wear resistance performance.

**Table 1:** IN718 composites reinforced with various amounts of BN and their addition effects on relative density and nitrogen amount.

	IN718 (vol%)	BN (vol%)	Theoretical density (g/cm^3^)	Measured density (g/cm^3^)	Nitrogen amount (wt%)
**A**	100.0	0.0	8.19	8.12	0.1833
**B**	94.0	6.0	7.82	7.68	4.1973
**C**	88.0	12.0	7.46	7.03	9.3456
**O**	0.0	100.0	2.29	—	53.9841

## Conclusions

A LPBF treatment was beneficial for fabrication of IN718 composites reinforced with BN. The results of microscopic, spectroscopic, and mechanical analyses of the SLM-built MMCs confirmed that IN718 was successfully reinforced with BN. Initially, the SEM observations indicated the morphological transformation of the composites according to the distribution of the reinforcement in the matrix. Qualitative analysis via TEM revealed that the BN reinforcement resulted in substantial incorporation within the IN718 matrix. Spectroscopic investigations (XRD and XPS) confirmed that no chemical reaction occurred between BN and IN718, despite the use of a high-power laser and consecutive rapid solidifications. In addition, the thermal behavior of the MMCs indicated by TGA–DSC analyses, and determined using another spectroscopic method, demonstrated that incorporation of BN within IN718 improved the heat resistance of the composites, dampening the sharpness of the melting temperature peak at 1364 °C. Furthermore, the specific hardness and compressive strength of the IN718 composite reinforced with 12 vol% BN increased (41.7 Hv_0.5_ **·**cm^3^/g and 92.4 MPa**·**cm^3^/g, respectively) compared with those of conventional IN718 alloy (38.7 Hv_0.5_ **·**cm^3^/g and 89.4 MPa**·**cm^3^/g, respectively). Their gradual increases were caused by the addition of BN reinforcement with low density (2.29 g/cm^3^). Finally, the BN/IN718 composite achieved the lower COF (0.31 at 2400 s), compared to the standard IN718 alloy (0.43 at 2400 s). This was achieved due to sliding and lubricating effects based upon the exfoliating surface of BN, which increased the wear resistance of the composite.

## Materials and Methods

### Preparation of gas-atomized IN718 micropowders and ball-milled h-BN nanosheets and application of the LPBF technique

Gas-atomized IN718 powders with particle size in the range 10–45 µm were used in this study. An IN718 ingot was obtained from a supplier (SeAH Special Steel, Republic of Korea). The IN718 powders were fabricated using a gas atomizer (Hot Gas Atomization System, PSI Ltd., UK)^48,49,59^. For the gas atomization process, 2.0 kg of IN718 ingot was melted in a graphite crucible heated to 1600 °C for 30 min. The molten IN718 liquid was then atomized to form small droplets that were rapidly solidified in a gas flow at 30 bar. The collected gas‐atomized IN718 powders were passed through a series of standard sieves (American Society for Testing and Materials, ASTM E11) to obtain IN718 powder particles of the intended size. Commercially available BN (98% purity) in the form of nanosheets with a hexagonal close-packed crystal structure and average diameter <1 µm was obtained from Sigma Aldrich and once obtained, was transferred into a stainless steel bowl. The diameter of the stainless steel balls used in the horizontal planetary mill (ARE-310, Dongwhan, Republic of Korea) was 10.3 mm, and the milling occurred at 150 r/min for 24 h^28^. The ball-to-powder ratio was 50:1 and 15 mL of isopropyl alcohol was used as solvent^28^. After the planetary ball-milling process, the product was further cleaned using sonication for 1 h in isopropyl alcohol. The BN nanosheets were subsequently dried at 80 °C for one day.

The BN nanosheets were mixed with the IN718 micropowder, and each mixture (as shown in Table 1) was obtained by combining IN718 micropowder with 0, 6, or 12 vol% BN nanosheets and using a tubular shaker mixer (CH-4005, Willy A. Bachofen AG Maschinenfabrik, Switzerland). The obtained BN/IN718 nanosheet-micropowder mixture was then subjected to the LPBF using a metal powder laser melting system (Concept Mlab LaserCusing, Germany). Cuboid specimens of IN718 alloy and the BN/IN718 composites (10 × 10 × 10 mm^3^) were manufactured using a 90 W laser, layer thickness of 25 μm, hatch spacing of 80 μm, and laser scanning speed of 800 mm/s. All specimens were prepared using a Z-increment with the continuous line-scanning strategy, under a high-purity argon gas mixture (<0.3% oxygen). Afterwards to study their microstructure, the samples were etched using a solution containing chloric acid, nitric acid, and deionized water (3.0: 1.0: 4.0 vol%), respectively.

### Materials Characterization

The amount of each element initially present in the IN718 powders was measured using inductively coupled plasma–atomic emission spectroscopy (ICP–AES, Optima 7300DV, PerkinElmer, USA). The concentration of oxygen and nitrogen in the IN718 (and in the BN nanosheets) was determined using an O/N analyzer (ON–900, Eltra GmbH, Germany)^48,49,59^. The density of the samples was measured using the Archimedes method. The surface morphology of the precursor micropowders and nanosheets, and of the alloy and composite products, was studied using a scanning electron microscope (SEM, JSM–5800, JEOL, Japan). The particle size in the powders was analyzed using a particle-size analyzer (LS13 320, Beckman Coulter Inc., USA) that included a laser-induced scattering measurement detector. Electron diffraction patterns and elemental composition were obtained using selective area electron diffraction (SAED) and X-ray electron diffraction spectroscopy (EDS) on a transmission electron microscope (TEM, JEM–ARM200F, JEOL, Japan). The crystal structure of the micropowders, nanosheets, alloy, and composites was determined using an X-ray diffractometer (XRD, D/Max–2500VL/PC, Rigaku International Corp., Japan) at 40 kV and 250 mA over the angular range (2θ) 20–80°. An X-ray photoelectron spectrometer (XPS, Quantera SXM, ULVAC–PHI, Japan) and XPSpeak 4.1 software were used to obtain more detailed information about the compounds in the BN-reinforced IN718 composite and the BN nanosheets. The Vickers hardness of the alloy and composites was measured using a hardness tester (HM–211, Mitutoyo, Japan) at 5.0 N loading and 12 s indentation time. The compressive test was conducted using a universal compression tester (2000KPX, Instron, USA) with a load of 2000 kN. The thermal behavior of the BN nanosheets, IN718 alloy, and BN/IN718 composites was measured using a differential scanning calorimeter (DSC, Q600, TA Instruments, USA) under an Ar gas flow while heating at 10 K/min. A dry sliding-wear test was conducted in a ball-on-disk tribometer (Ball/Pin on Disc, J&L Tech Co., Ltd., Republic of Korea) in air at room temperature. The surfaces of the specimens were polished prior to the wear test. A tungsten carbide ball with a diameter of 5.4 mm was used as the counterface material under a test load of 5.0 N. The friction unit was rotated at 200 r/min for 2400 s. The coefficient of friction (COF) of the specimens was recorded during the wear test.

## Electronic supplementary material

Supplementary Document

LPBF 1

LPBF 2

WEAR TEST
