# Supplementary material for: Thermo-mechanical improvement of Inconel 718 using ex situ boron nitride-reinforced composites processed by laser powder bed fusion
Source: Sci Rep. 2017 Oct 30;7:14359. doi: 10.1038/s41598-017-14713-1 (PMC5662723; doi:10.1038/s41598-017-14713-1)
Supplement: Supplementary file 1 — Supplementary Document [file 41598_2017_14713_MOESM1_ESM.doc]

**Thermo-mechanical improvement of Inconel 718 using *ex situ* boron nitride-reinforced composites processed by laser powder bed fusion**

Sang Hoon Kim^1,a^, Gi-Hun Shin^2,a^, Byoung-Kee Kim^2^, Kyung Tae Kim^1^, Dong-Yeol Yang^1^, Clodualdo Aranas Jr.^3^, Joon-Phil Choi^3,*^, and Ji-Hun Yu^1,*^

^1^Powder Technology Department, Korea Institute of Materials Science, Changwon 51508, Republic of Korea

^2^Department of Materials Science and Engineering, University of Ulsan, Ulsan 44610, Republic of Korea

^3^Department of Mining and Materials Engineering, McGill University, 3610 University Street, Montreal, QC, H3A 0C5, Canada

^a^Two authors contributed equally and should be considered co-first authors.

^*^Corresponding author: joonphil.choi@mail.mcgill.ca (Joon-Phil Choi), jhyu01@kims.re.kr (Ji-Hun Yu)

With regard to a casting method to fabricate a composite that consists of constituents with different relative density and thermal diffusivity, and particularly when using a ceramic reinforcement material in a metal matrix material, severe deformation may occur and can induce mechanical defects at the interfacial boundaries between the two dissimilar materials (reinforcement and matrix)^1-4^. This method also results in localized distribution of the reinforcement material within the matrix material, which can result in mechanical degradation^1-4^. In fact, achieving high strength and homogeneous distribution of the reinforcement in the matrix is the main issue in the fabrication of a composite. This is even true when using energy-effective solid-state diffusion processes (e.g., powder injection molding or hot isostatic pressing)^5, 6^. For example, the highly useful powder injection molding method is used to overcome such issues, providing more uniform distribution of the reinforcement in the matrix. Even so, fabrication of the ceramic-reinforced MMCs is still difficult due to formation collapse caused by difference in their respective thermal diffusivity, or to structural fracture along the interfacial boundaries between the ceramic and the metal^5^. It is also possible that high thermal stress and residual mechanical strain are retained in such a structural component due to insufficient bonding between the reinforcement and the matrix^6^. Comparatively, laser beam irradiation with high thermal energy can reduce the potential for occurrence of such setbacks, particularly by controlling the time and speed of the laser. In addition, a vigorous blending process should be used to create a mixture of reinforcement and matrix materials with homogeneous distribution because uniform dispersion of reinforcement within the matrix can effectively provide any envisaged properties of the composites. The BN reinforcement has very low relative density and high thermal stability (compared to the IN718 matrix), making it difficult to use as reinforcement in the matrix due to its widely different physical properties. However, the use of an active blending process and *ex situ* LPBF technique with BN and IN718 can provide homogeneous morphology and strong bonding between the ceramic and the metal.

**References**

1. Hashim, J., Looney, L. & Hashmi, M. Metal matrix composites: production by the stir casting method. *J. Mater. Process. Technol.* **92**, 1-7 (1999).

2. Tjong, S. C. & Ma, Z. Microstructural and mechanical characteristics of in situ metal matrix composites. *Materials Science and Engineering: R: Reports* **29**, 49-113 (2000).

3. Tjong, S. C. Recent progress in the development and properties of novel metal matrix nanocomposites reinforced with carbon nanotubes and graphene nanosheets. *Materials Science and Engineering: R: Reports* **74**, 281-350 (2013).

4. LLorca, J. Fatigue of particle-and whisker-reinforced metal-matrix composites. *Progress in Materials Science* **47**, 283-353 (2002).

5. Imbaby, M. & Jiang, K. Fabrication of free standing 316-L stainless steel–Al_2_O_3_ composite micro machine parts by soft moulding. *Acta Materialia* **57**, 4751-4757 (2009).

6. Mao, X., Oh, K. H., Kang, S. H., Kim, T. K. & Jang, J. On the coherency of Y_2_Ti_2_O_7_ particles with austenitic matrix of oxide dispersion strengthened steel. *Acta Materialia* **89**, 141-152 (2015).


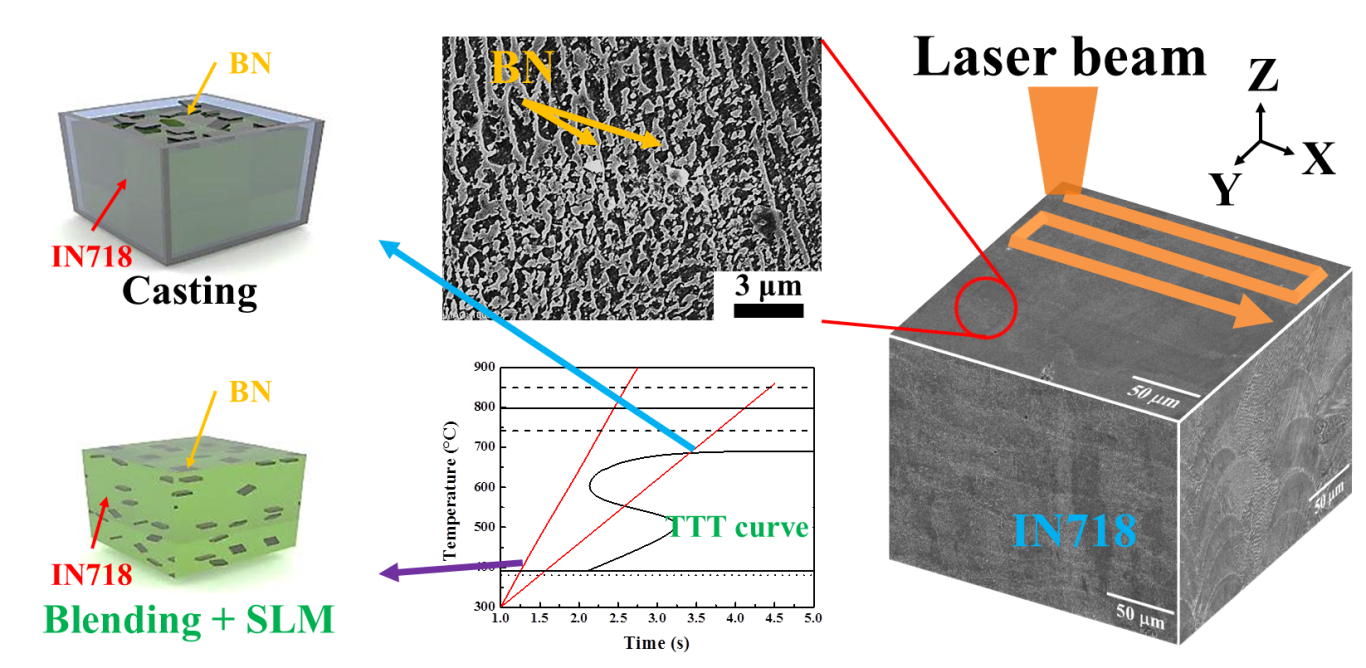


**Supplementary Figure 1.** BN-reinforced IN718 composites were fabricated using the SLM technique with a rapid solidification process. Thus, the BN was randomly distributed within the IN718 in the case of the SLM process, compared to the BN floated (locally distributed) upon the IN718 when cast.


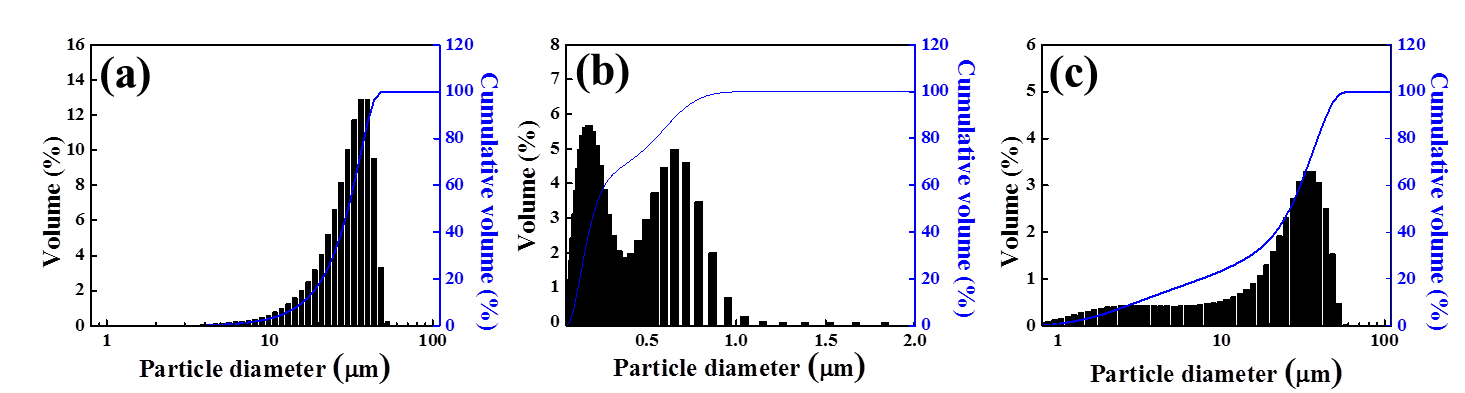


**Supplementary Figure 2.** Particle size distribution of (a) IN718 powders, (b) BN nanosheets, and (c) 12 vol% BN nanosheet + IN718 micropowder mixture.


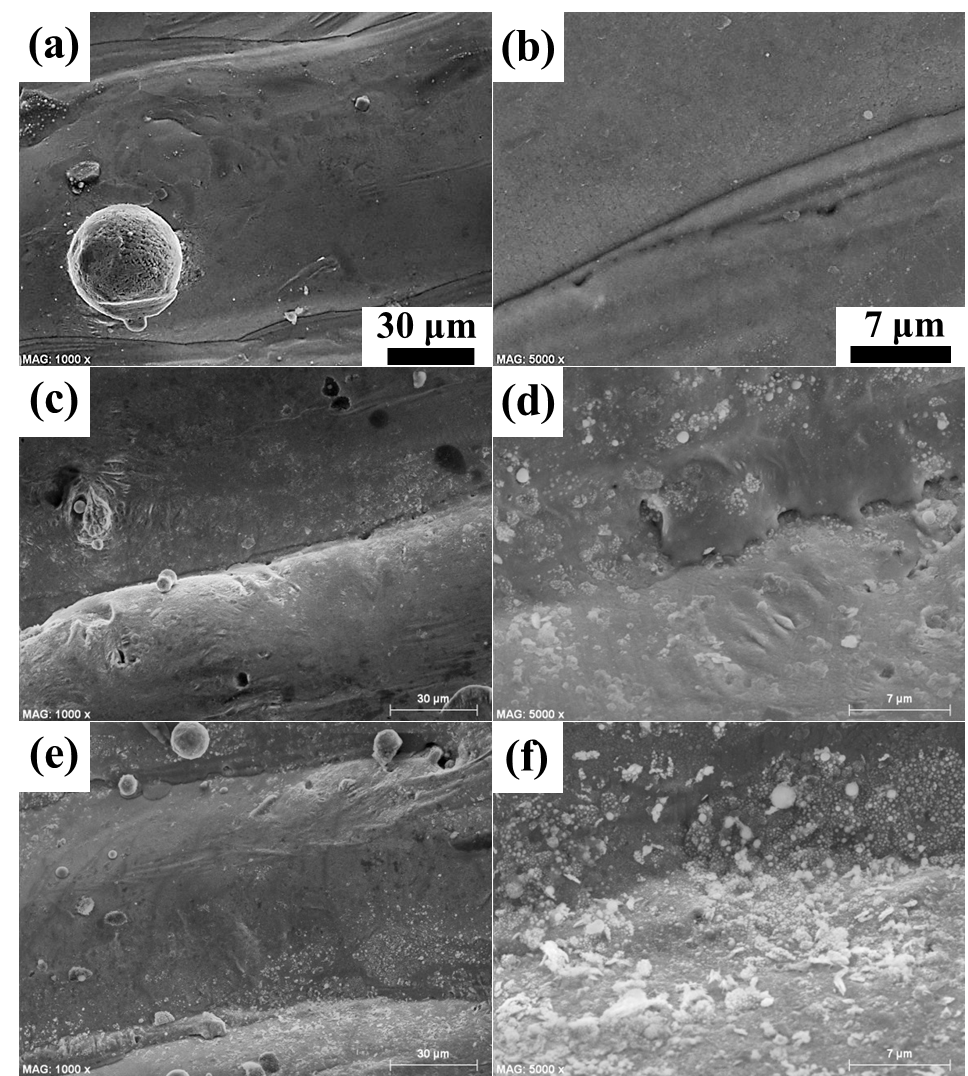


**Supplementary Figure 3.** SEM images of (a and b) IN718 alloy, (c and d) IN718 composite reinforced with 6 vol% BN, and (e and f) IN718 composite reinforced with 12 vol% BN after SLM.


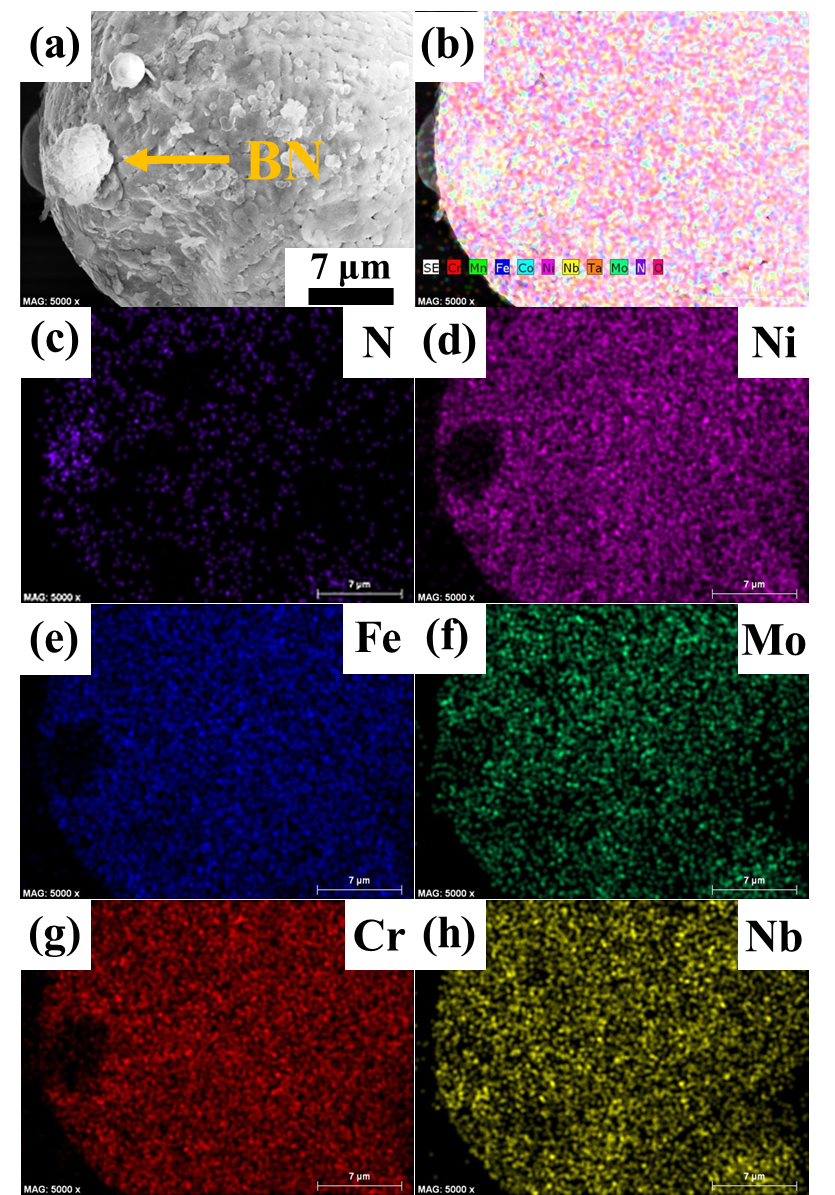


**Supplementary Figure 4.** SEM image of (a) 6 vol% BN nanosheet + IN718 micropowder mixture prior to SLM; (b) EDS map of (a); (c) Nitrogen, (d) Nickel, (e) Iron, (f) Molybdenum, (g) Chromium, and (h) Niobium.


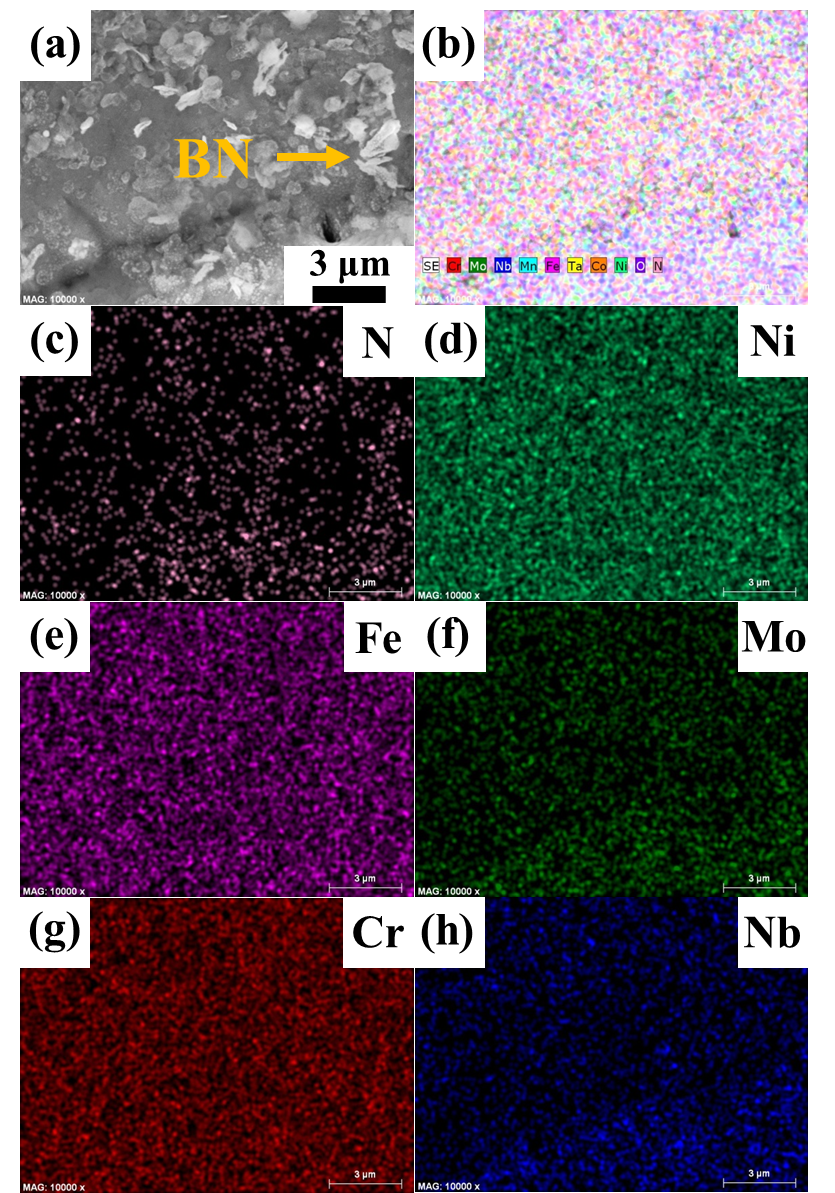


**Supplementary Figure 5.** SEM image of (a) IN718 composite reinforced with 12 vol% BN after SLM; (b) EDS map of (a); (c) Nitrogen, (d) Nickel, (e) Iron, (f) Molybdenum, (g) Chromium, and (h) Niobium.


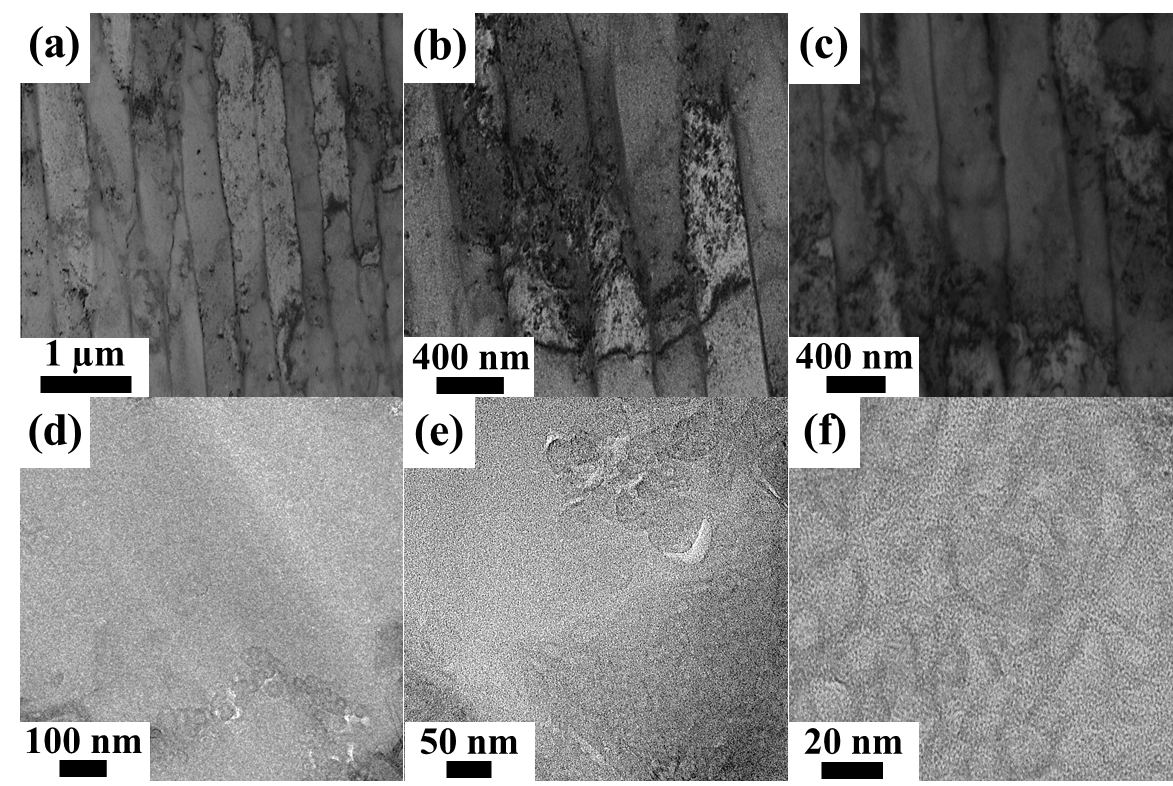


**Supplementary Figure 6.** TEM images at (a–c) IN718 region and (d–f) BN region of IN718 composite reinforced with 12 vol% BN after SLM.


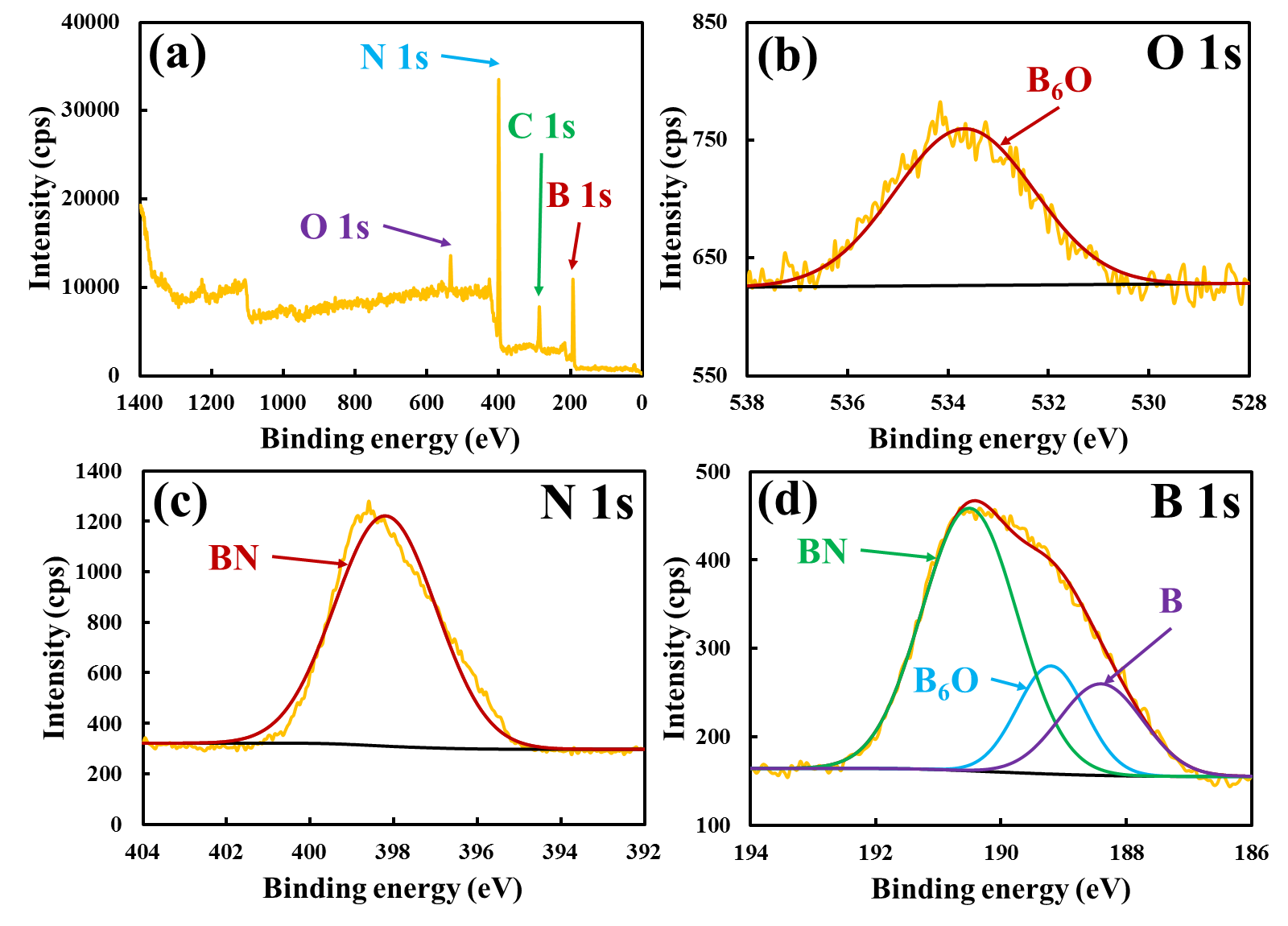


**Supplementary Figure 7.** XPS of BN nanosheets.


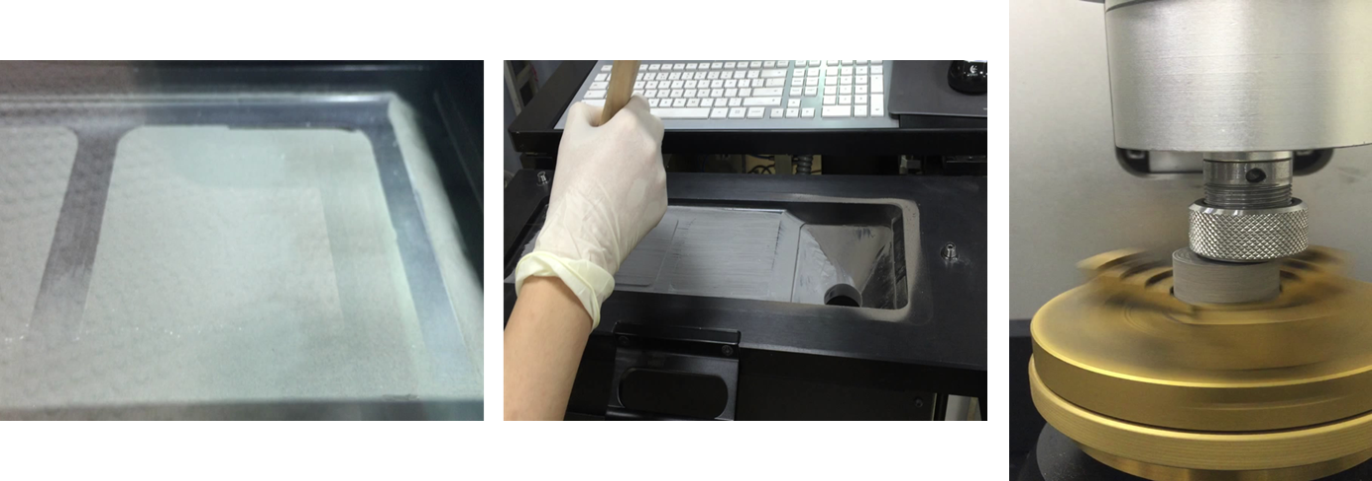


**Videos.** In situ video recordings of the powder bed fusion process for fabrication of IN718 composite reinforced with 6 vol% BN. A dry sliding wear test was conducted using a ball-on-disk tribometer in air at room temperature.

– The upper layer of the powder bed was directly consolidated with the bottom layer of the powder bed by SLM; then, the remaining powder was brushed and vacuumed into the used powder collector.

– Fabrication of IN718 composite reinforced with 6 vol% BN using the powder bed type of AM, to prepare the samples for the wear test.

– The samples prepared for the wear test were cylindrical (diameter 20 mm, thickness 10 mm).
